# Supplementary figures and images for: REM1.3's phospho-status defines its plasma membrane nanodomain organization and activity in restricting PVX cell-to-cell movement
Source: PLoS Pathog. 2018 Nov 12;14(11):e1007378. doi: 10.1371/journal.ppat.1007378 (PMC6258466; doi:10.1371/journal.ppat.1007378)

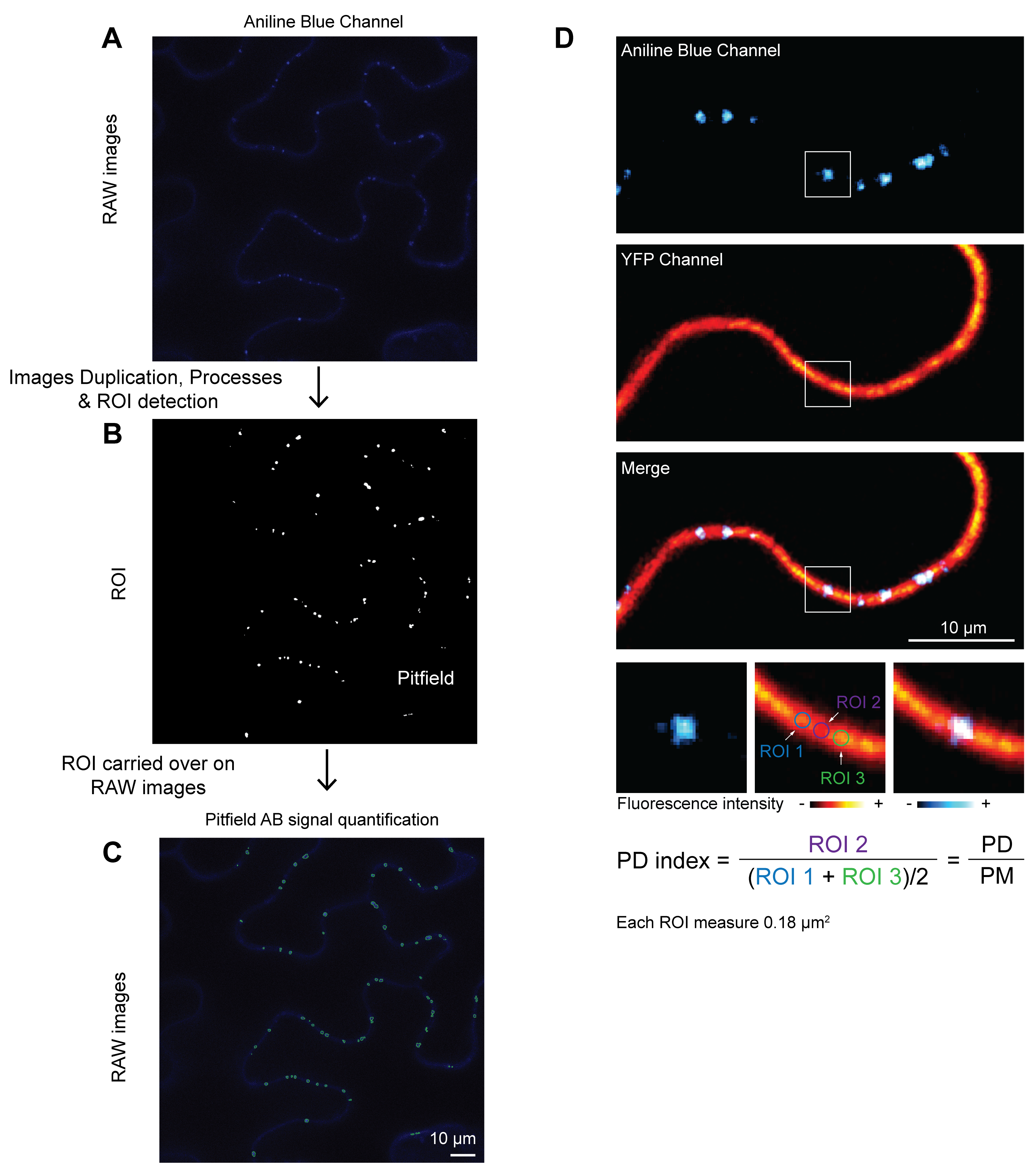

Supplement: S1 Fig — (A) Original sample image is an 8-bit, single-channel image. (B) Masks of total Region Of Interest (ROI) objects before particle analysis were created using the following filters; background subtraction with a rolling ball radius as in [43]; “smooth” twice and an auto-local threshold Max Entropy dark, creating a black and white mask, used for particle detection. (C) Overlay of outlines of the analyzed ROI (green; after particle analysis with particle size 3–100 pixel2 circularity (0.3–1) exclude on edge) with the original image. Scale bar indicates 10 μm. (D) Quantification of PD Index; after aniline blue labeled pit-field detection, YFP-REM1.3 fluorescence intensity was manually measured at pit-field level (ROI2) and surrounding PM (ROI1 and ROI3) using a circle of fixed area (0.18 μm2). The PD index was then calculated as the ratio between YFP-REM1.3 pit-field fluorescence (ROI2) and the mean of YFP-REM1.3 fluorescence intensity at surrounding PM (ROI1+ROI3). (TIF) [file ppat.1007378.s001.tif]

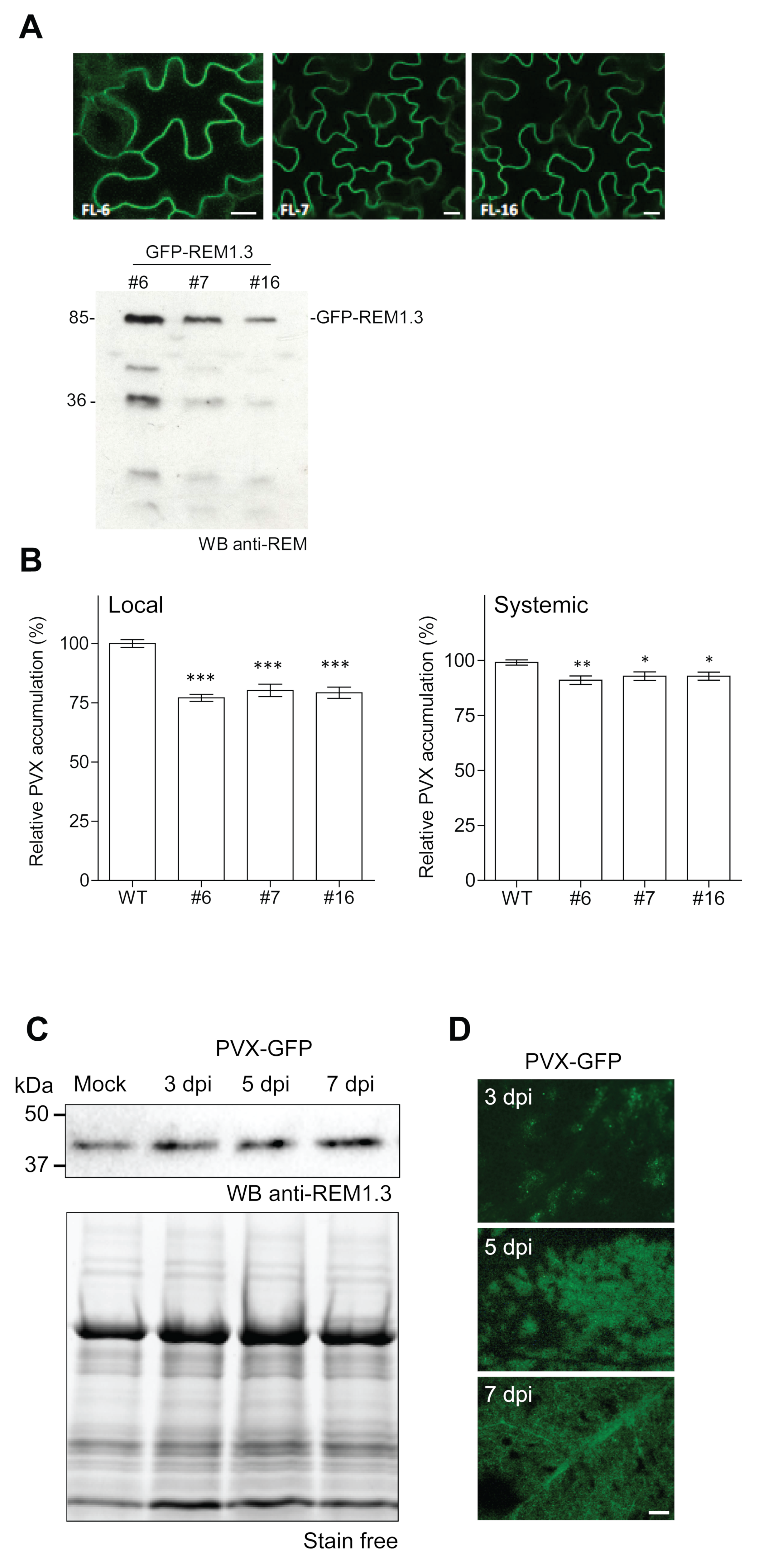

Supplement: S2 Fig — A. Top, Confocal image showing GFP-REM1.3 localisation in the PM in N. benthamiana lines Bottom, The GFP:REM1.3 expression in three independent transgenic lines #6, 7 and 16 was tested by Western blot against REM and showed that it contains at least three times the amount of endogenous N. benthamiana REM. B. PVX infection assays in independent stably expressing GFP-REM1.3 and wild-type control N. benthamiana lines. Viral charge was assayed by test DAS-ELISA using antibodies to PVX coat protein on distal (3 nodes above inoculation) leaves at 14 DAI. Three independent experiments were performed with five plants for each transgenic line and non-transgenic (WT). Error bars show SE, and significance is assessed by Dunnett’s multiple comparison test against WT (*, P < 0.1; **, P < 0.05; ***, P < 0.001). C, Western blot against REM1.3 was performed on total protein extracts from wild type N. benthamiana leaves infected by PVX-GFP at 0, 3, 5 and 7 DAI. Stain free loading is indicated below. D, Confocal images showing PVX-GFP foci at the indicated DAI, tested in C. (TIF) [file ppat.1007378.s002.tif]

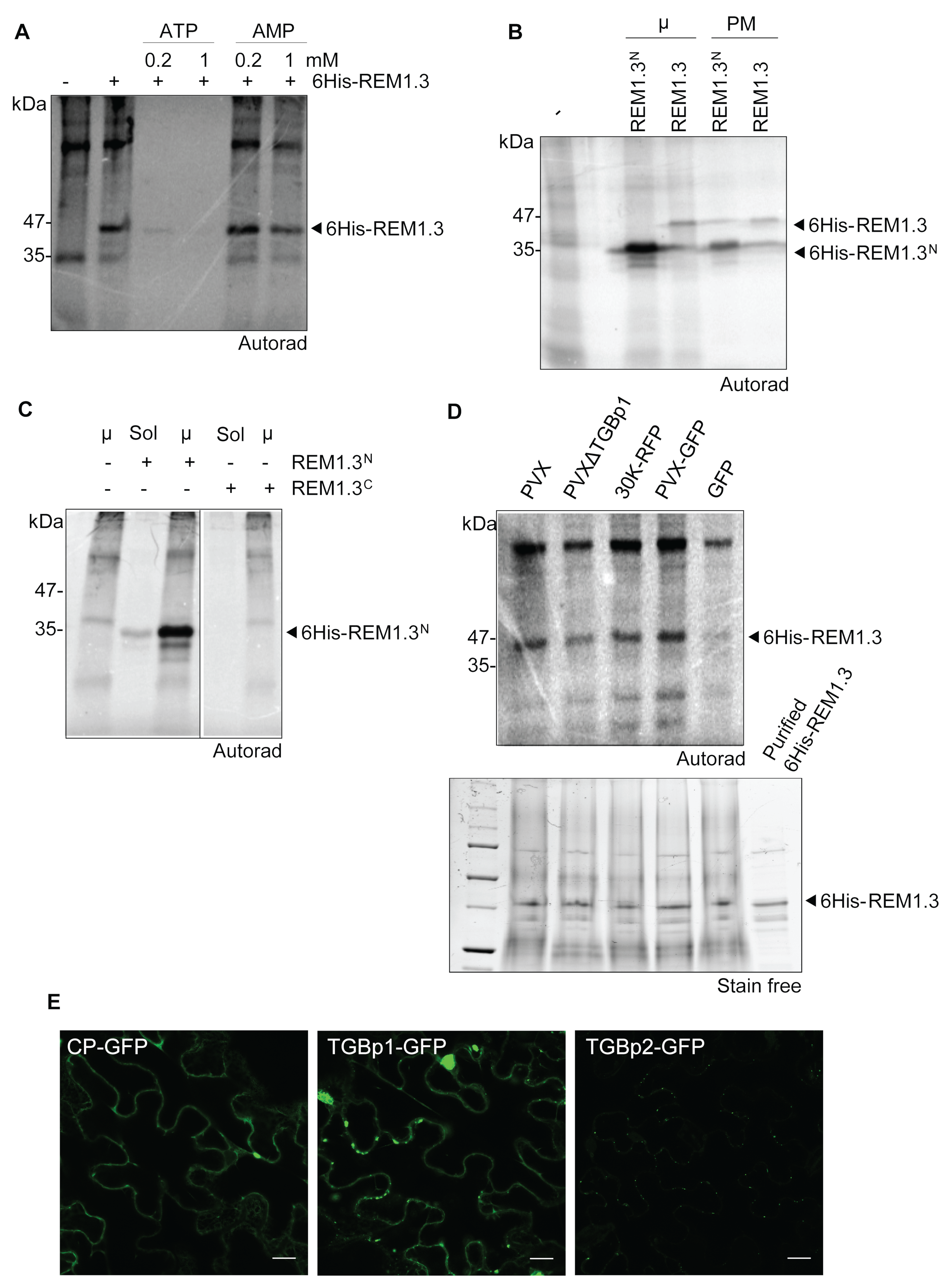

Supplement: S3 Fig — (A) Effect of the addition of ATP or AMP in in vitro phosphorylation assays of 6His-REM1.3 by kinase(s) in microsomal (μ) or PM extracts of N. benthamiana leaves developed by autoradiography. (B) 6His-REM1.3N and 6His-REM1.3 phosphorylation by healthy N. benthamiana leaf microsomal (μ) and plasma membrane (PM) extracts. (C) 6His-REM1.3N and 6His-REM1.3C phosphorylation by kinase(s) in microsomal (μ) and soluble extracts. (D) 6His-REM1.3 was differentially phosphorylated by leaf microsomal extracts expressing the indicated constructs i.e. PVX alone, PVX deleted for TGBp1 (PVXΔTGBp1), 30K protein from Tobacco Mosaic Virus (TMV), PVX fused to GFP, and GFP alone at 4 DAI. See the rationale Fig 2E. Control of loading is shown after stain free procedure. In all phosphorylation experiments about 10μg of total protein extracts and 1μg of affinity purified 6His-REM1.3, REM1.3N or REM1.3C were loaded per lane. (E) Controls of expression of fluorescently-tagged viral proteins, namely CP, TGBp1, TGBp2 used in Fig 2. (TIF) [file ppat.1007378.s003.tif]

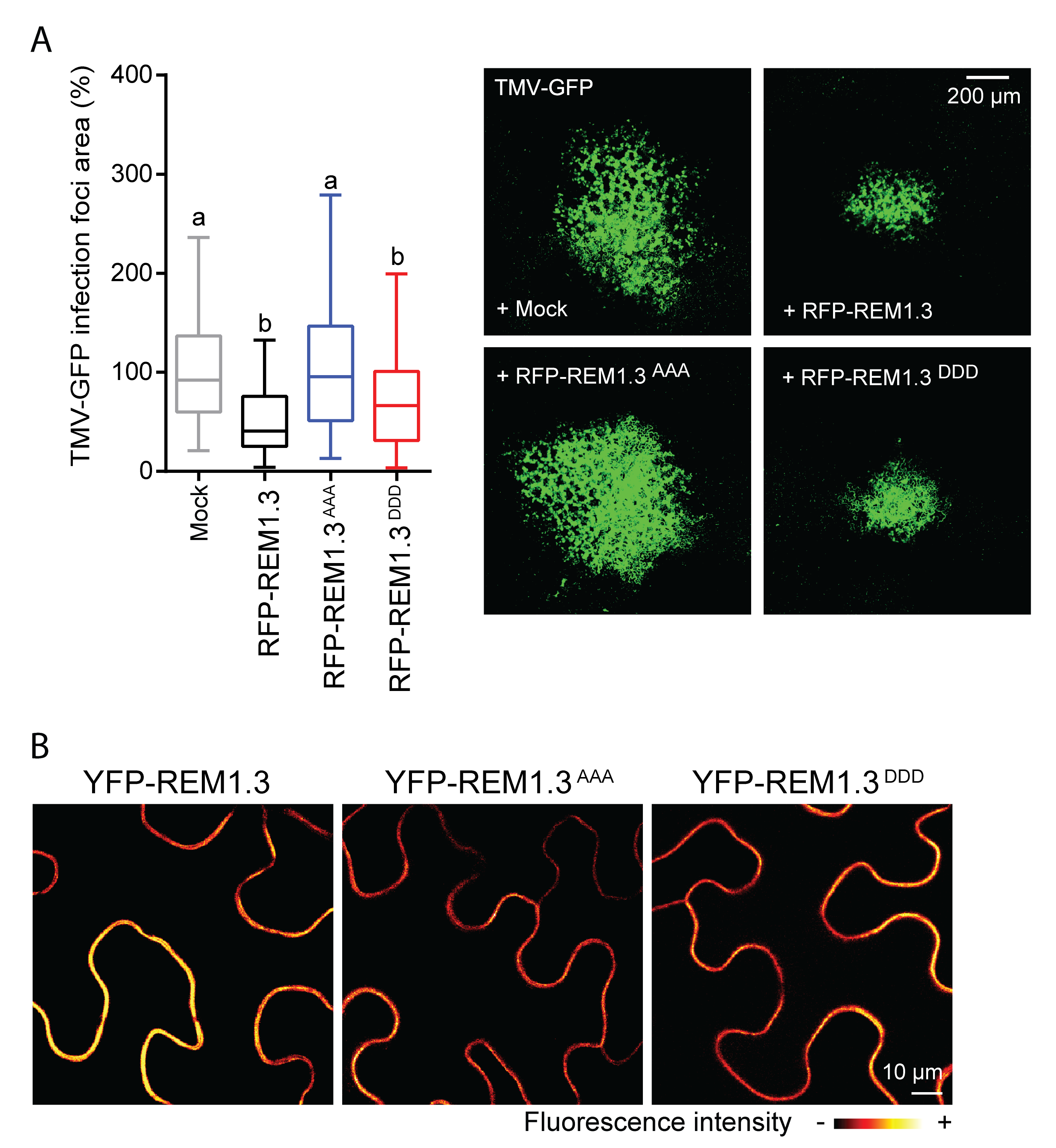

Supplement: S4 Fig — (A) Representative epifluorescence microscopy images of Tobacco Mosaic Virus (TMV-GFP) infection foci in N. benthamiana leaf epidermal cells at 5 DAI. Graph represents the relative foci area of REM1.3 or phosphomutants (S74, T86 and S91 into Alanine, AAA or Aspartic Acid, DDD) compared to mock control (co-infiltration of PVX-GFP with an empty A. tumefaciens strain). About 78–128 foci per condition were measured in 2 independent biological repeats. Dunn’s multiple comparison tests were applied for statistical analysis, p<0.001. (B) Confocal microscopy images of secant views of N. benthamiana epidermal cells expressing YFP-REM1.3, YFP-REM1.3AAA and YFP-REM1.3DDD at 2 DAI. Scale bar indicates 10 μm. (TIF) [file ppat.1007378.s004.tif]

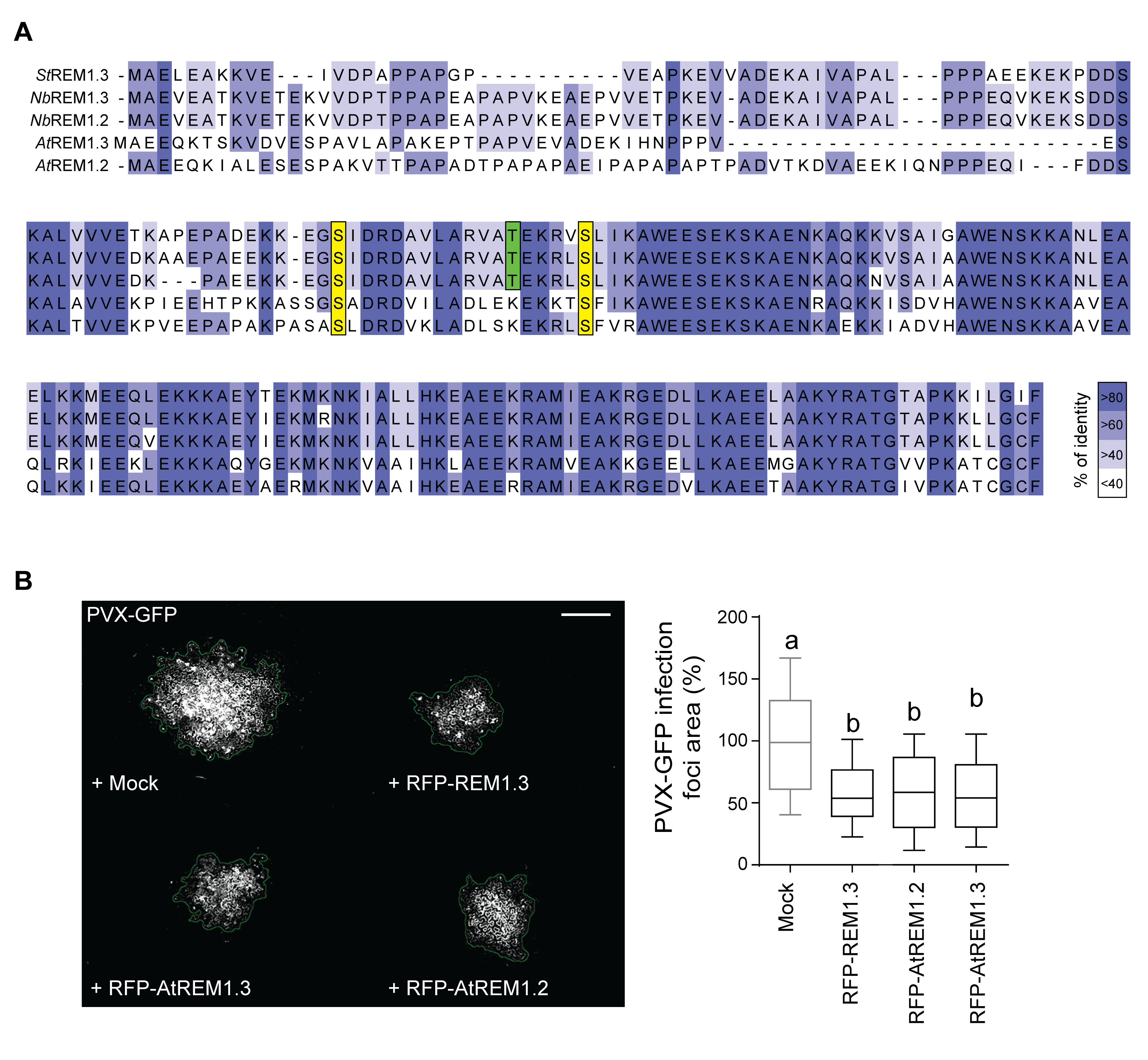

Supplement: S5 Fig — (A) Clustal alignments of protein sequences of group 1b REMORINs: AtREM1.2, AtREM1.3, NbREM1.2, NbREM1.3 and REM1.3 (StREM1.3). Blue color-coding shows percentage of identity. The REM1.3 S74, T81 and S91 sites are highlighted. (B) Left, Representative epifluorescence microscopy images of PVX-GFP infection foci on N. benthamiana leaf epidermal cells transiently expressing RFP-REM1.3, RFP-AtREM1.2 or RFP-AtREM1.3 at 5 DAI. Scale bar indicate 400 μm. Right, Graph represents the relative PVX-GFP infection foci area in the presence of RFP-REM1.3 or Arabidopsis homologs compared to mock control (co-infiltration of PVX-GFP with empty A. tumefaciens strain). At least 184 foci per condition in 4 independent biological repeats were measured. Statistical differences are indicated by letters as revealed by Dunn’s multiple comparisons test p<0.001. (TIF) [file ppat.1007378.s005.tif]

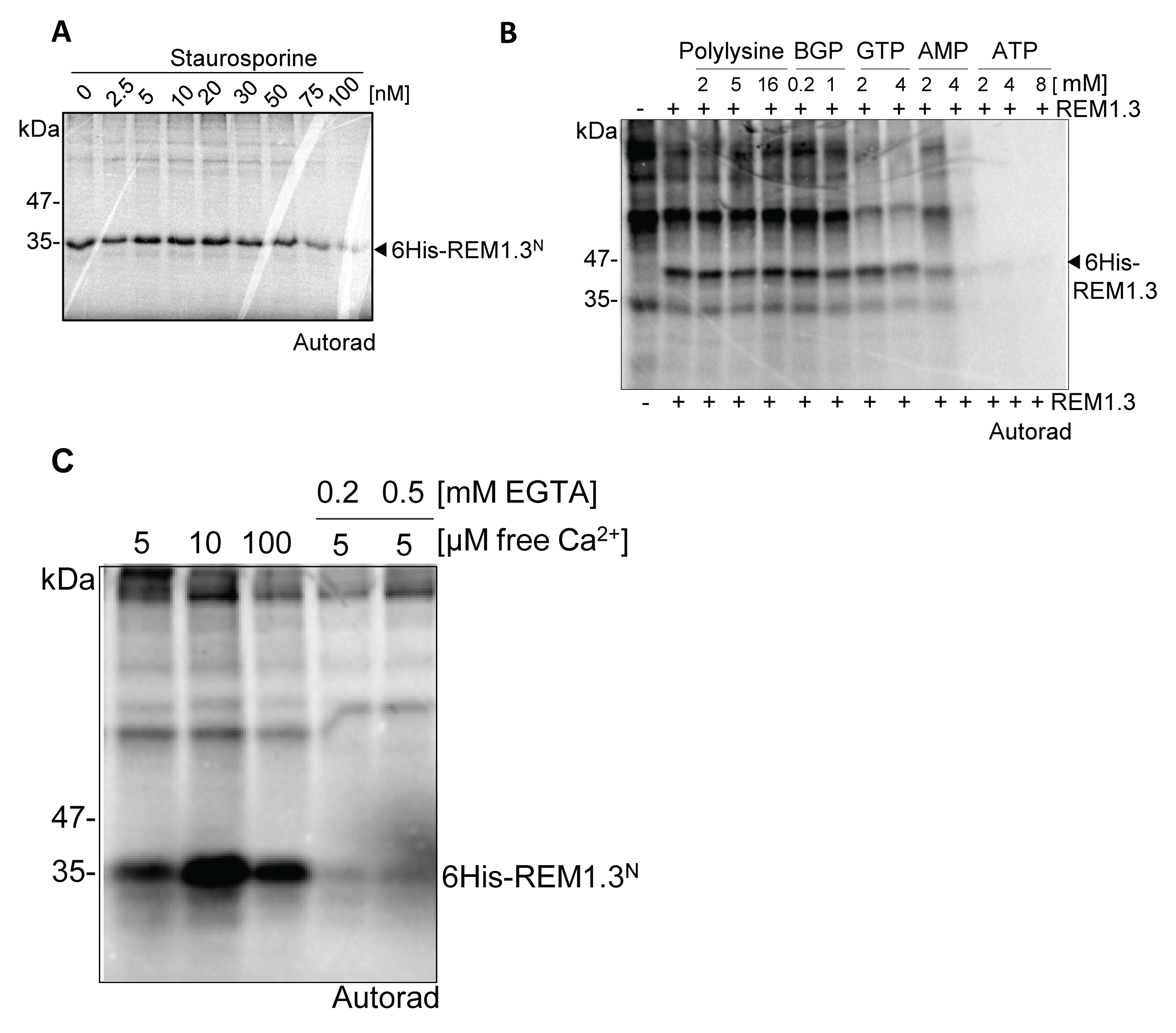

Supplement: S6 Fig — Autoradiography reveals in vitro phosphorylated 6His-REM1.3N (A) or 6His-REM1.3 (B) by microsomal extracts of healthy N. benthamiana leaves in the presence of increasing concentrations of staurosporine (A) or Polylysine, β-glycerophosphate (BGP), GTP, AMP and ATP (B). (C) Effect of Ca2+ and EGTA on 6His-REM1.3N phosphorylation by kinase(s) in microsomal extracts. (TIF) [file ppat.1007378.s006.tif]

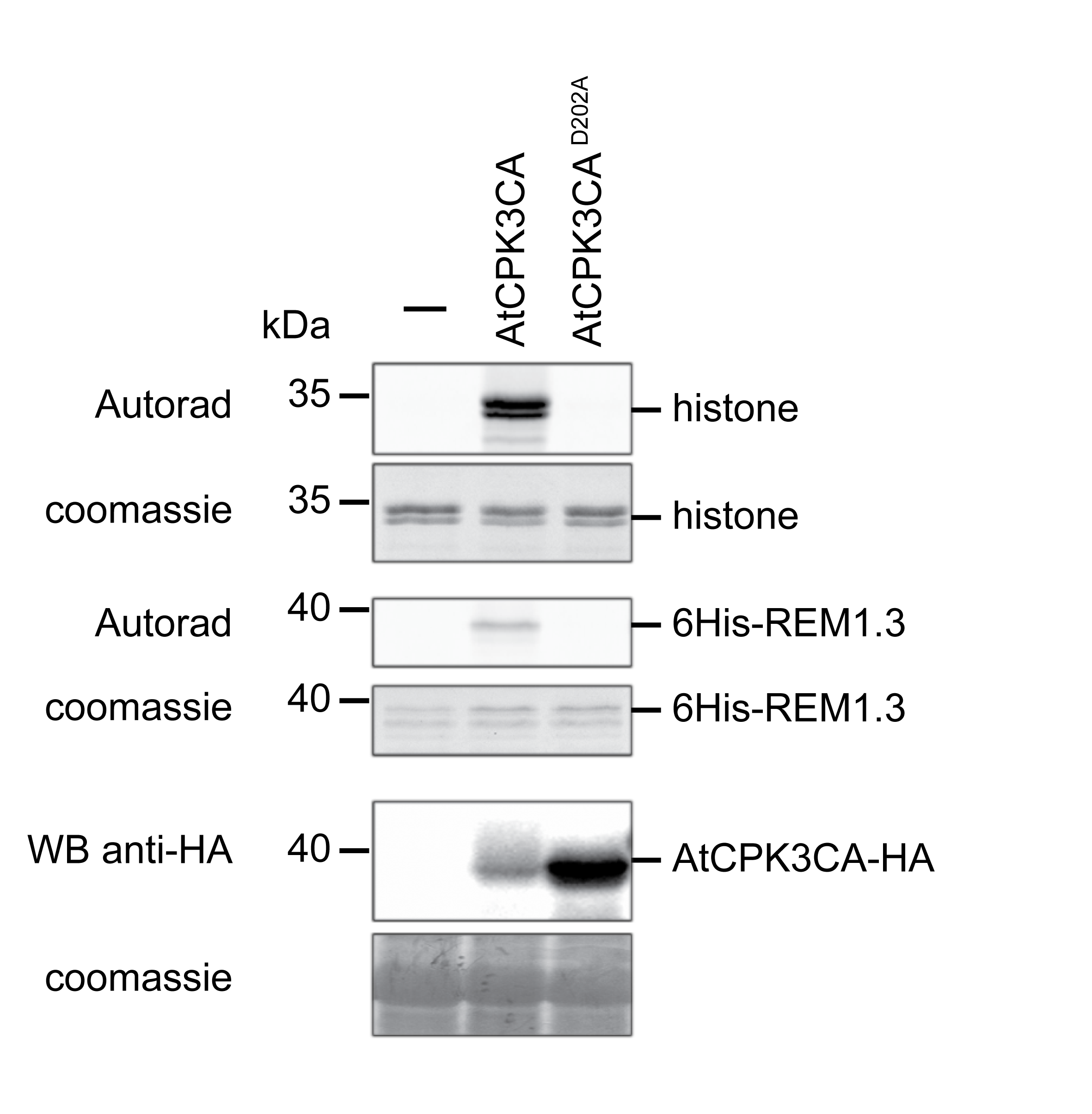

Supplement: S7 Fig — AtCPK3CA-HA and AtCPK3CAD202A-HA were expressed in Arabidopsis thaliana mesophyll protoplasts. Immunoprecipitated proteins were incubated with ATP [γ-33P] and submitted to an in vitro kinase assay using 6His-REM1.3 or histone as substrates. In vitro kinase assays were revealed by autoradiography. Trans-phosphorylation of the substrates 6His-REM1.3 or histone is indicated. Western blot against HA shows the expression levels of the expressed proteins. (TIF) [file ppat.1007378.s007.tif]

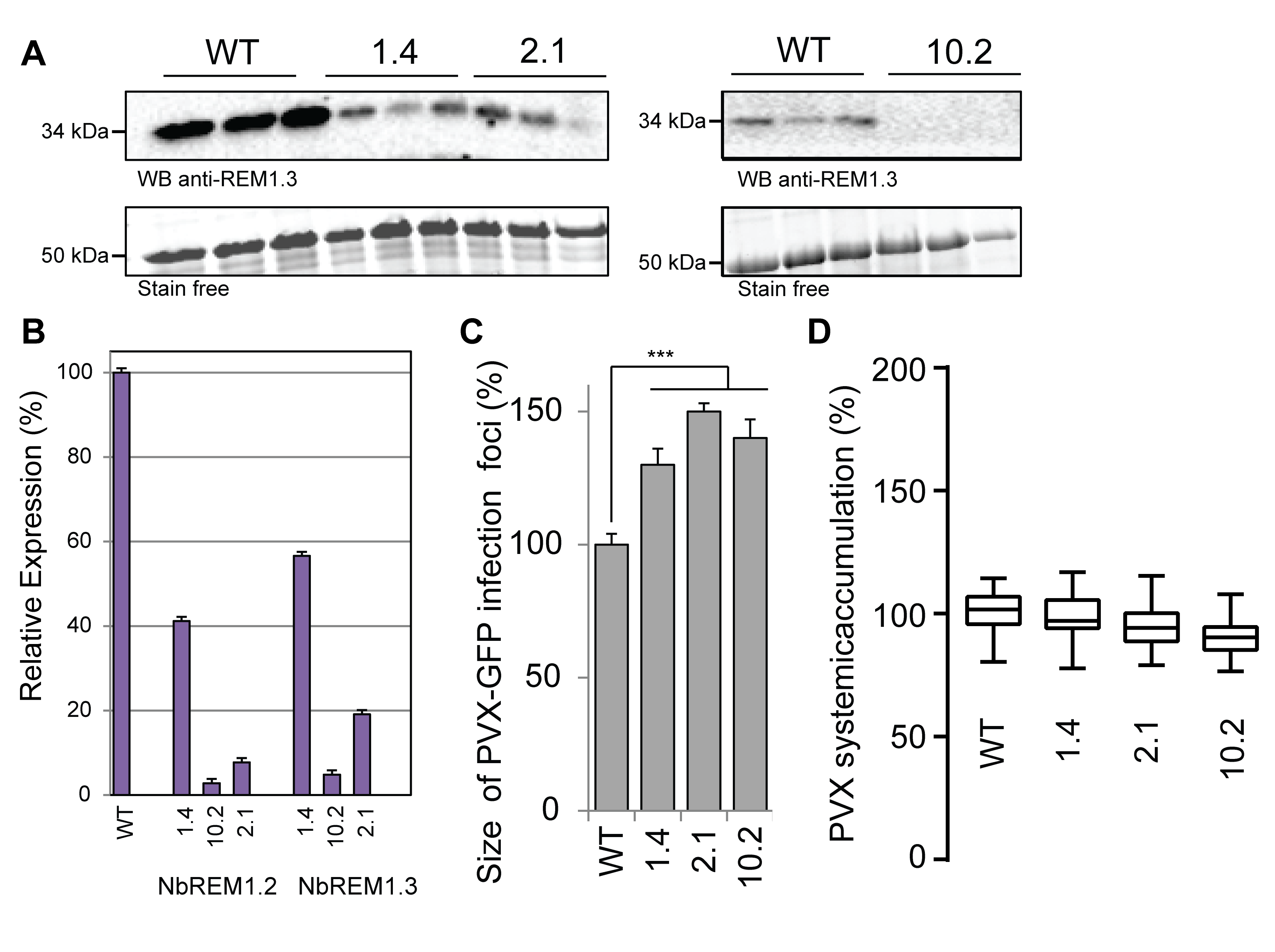

Supplement: S8 Fig — (A) Protein expression levels of endogenous NbREMs in the hpREM lines, determined by Western Blot analysis using anti-REM1.3 antibodies. Protein extracts from three independent plants per line were used, namely lines 1.4, 2.1, 10.2. (B) Expression of endogenous NbREMs in the hpREM lines determined by RT-qPCR analysis. Results are expressed relative to the NbREMs expression levels in the WT background. RT-qPCR signals were normalized to actin levels. (C) PVX-GFP spreading is accelerated in the hpREM lines. Graph represents the PVX-GFP infection foci area in the different hpREM lines compared to WT. At least three independent experiments were performed. Error bars show +/- SEM. Statistical differences compared to WT were determined by Mann- Whitney test *** p<0.001. (D) PVX systemic propagation is inversely correlated with REM levels in 4-week-old transgenic N. benthamiana leaves. Viral charges were assayed by DAS ELISA using antibodies to PVX coat protein (diluted on 1/100) on distal leaves (at 3 nodes above the inoculated leaves) at 10 DAI. 3 independent experiments were performed with eight plants for each hpREM transgenic line and WT or empty vector control (mock). Error bars show SE, and significance is assessed by Mann-Whitney non-parametric test (*, P < 0.1; **, P < 0.05; ***, P < 0.001). (TIF) [file ppat.1007378.s008.tif]
